# Duration of nivolumab for pretreated, advanced non–small‐cell lung cancer

**DOI:** 10.1002/cam4.3120

**Published:** 2020-05-15

**Authors:** Margaux Geier, Renaud Descourt, Romain Corre, Guillaume Léveiller, Régine Lamy, Éric Goarant, Jean‐Louis Bizec, Cyril Bernier, Gilles Quéré, Karim Amrane, Elisabeth Gaye, François Lucia, Emilie Burte, Christos Chouaid, Gilles Robinet

**Affiliations:** ^1^ Department of Oncology CHRU Morvan University Hospital of Brest Brest France; ^2^ Department of Pulmonary Diseases CHU Pont‐Chaillou University Hospital of Rennes Rennes France; ^3^ Department of Pulmonary Diseases CH Yves Le Foll Hospital of Saint‐Brieuc Saint‐Brieuc France; ^4^ Department of Oncology CH Bretagne‐Sud, Hospital of Lorient Lorient France; ^5^ Department of Pulmonary Diseases Hospital of Saint‐Malo Saint‐Malo France; ^6^ Department of Pulmonary Diseases CH Bretagne‐Atlantique, Hospital of Vannes Vannes France; ^7^ Department of Pulmonary Diseases CH Rene‐Pleven, Hospital of Dinan Dinan France; ^8^ Department of Pulmonary Diseases Hospital of Morlaix Morlaix France; ^9^ eXYSTAT on behalf of the ABCT Association Malakoff France; ^10^ Department of Pulmonary Diseases CHI Créteil Créteil France

**Keywords:** immunotherapy, long‐term survivors, nivolumab, non–small‐cell lung cancer, real‐life, treatment duration

## Abstract

**Background:**

A standard of care for pretreated, advanced non–small‐cell lung cancers (NSCLCs), nivolumab has demonstrated long‐term benefit when administered for 2 years. We aimed to better discern an optimized administration duration by retrospectively analyzing real‐life long‐term efficacy in a prospective cohort.

**Methods:**

All nivolumab‐treated adults with advanced NSCLCs (01/09/2015 to 30/09/2016) from nine French centers were eligible. On 31/12/2018, patients who are alive ≥ 2 years after starting nivolumab were defined as long‐term survivors (LTSs) and were divided into three nivolumab treatment groups: <2, 2, or > 2 years. Co‐primary endpoints were LTSs’ progression‐free survival (PFS) and overall survival (OS).

**Results:**

The median follow‐up was 32 months (95% CI, 31.0 to 34.0). The 3‐year OS rate for the 259 cohort patients was 16.6%. Among them, 65 were LTSs: 47 treated < 2 years, 7 for 2 years, and 11 > 2 years. Their respective characteristics were: median age: 59, 52, and 58 years; smoking history: 92.9, 100, and 100%; adenocarcinomas: 66, 57.1, and 54.5%. LTSs’ median (m)PFS was 28.4 months; mOS was not reached. LTSs’ objective response rate was 61.6%. mOS was 32.7 months for those treated < 2 years and not reached for the others. The > 2‐year group's 3‐year OS was longer. Twenty‐eight LTSs experienced no disease progression; 7 had durable complete responses. However, LTSs had more frequent and more severe adverse events.

**Conclusion:**

In real‐life, prolonged nivolumab use provided long‐term benefit with 16.6% 3‐year OS and 25% LTSs. Survival tended to be prolonged with nivolumab continued beyond 2 years. Prospective randomized trials with adequate design are needed.

## Graphical abstract

Phase III trials didn't provide an optimal duration of immunotherapy for responder or stable patients in advanced non‐small‐cell lung cancer. A 2 years‐fixed duration or long‐term therapy are proposed to clinicians. The results of this observational study of 65 long‐term survivor pretreated patients showed a trends toward improvement of survival outcomes when continuing nivolumab after two years.

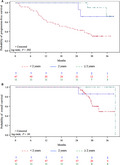

**Abbreviations**
AEs: adverse events
CR: complete response
CTCAE: Cancer Institute Common Terminology Criteria for Adverse Events
DP: disease progression
ICIs: immune‐checkpoint inhibitors
LTSs: long‐term survivors
mOS: median overall survival
mPFS: median progression‐free survival
NSCLC: non–small‐cell lung cancer
ORR: Objective response rate
PR: partial response

## 1 INTRODUCTION

Immune‐checkpoint inhibitors (ICIs) are the standard of care for locally advanced or metastatic non–small‐cell lung cancers (NSCLCs) that progressed after platinum‐based first‐line chemotherapy.^1^, ^2^ Pembrolizumab (anti‐programed cell‐death protein‐1 ligand‐1 (PD‐L1)) is indicated for patients with ≥ 1% tumor cells expressing PD‐L1; nivolumab and atezolizumab (directed against PD‐1) are indicated for all comers.^3^, ^4^ More recently, pembrolizumab monotherapy was approved as first‐line therapy for patients with previously untreated, advanced NSCLCs and a PD‐L1 tumor‐proportion score ≥ 50%.^5^ Immune‐checkpoint inhibitors offer a new paradigm of treatment options, obtaining improved objective response rates (ORR) and overall survival (OS), with satisfactory safety profiles compared to chemotherapy. The promise of immunotherapy has held‐up in real life, based on the findings of international cohort studies,^6^, ^7^, ^8^, ^9^, ^10^, ^11^, ^12^, ^13^ with the emergence of long‐term survivors (LTSs). Randomized, phase III trial results indicated 23% 2‐year OS for squamous NSCLCs^1^ and 29% for nonsquamous NSCLCs^2^ with nivolumab,^14^ and 31% with atezolizumab.^15^ The 2‐year OS with pembrolizumab was 30.1% for patients with PD‐L1 tumor‐proportion scores ≥ 1%^16^; patients with PD‐L1 ≥ 50% achieved 35% 36‐month OS.^17^

However, the optimal durations of first‐ and second‐line ICI administration remain unknown. Although clinical trial results demonstrated that second‐line ICIs provided long‐term benefit when administered for at least 2 years,^17^, ^18^ most survival data were based on patients receiving treatment until disease progression (DP) or unacceptable toxicity.

With longer follow‐up of a real‐life observational cohort, we attempted to better discern an optimized nivolumab administration duration by analyzing LTSs’ survival.

## 2 PATIENTS AND METHODS

### 2.1 Study population and procedures

IMMUNOBZH is a multicenter, noninterventional, cohort study conducted in nine French centers throughout the Brittany region.^13^ The cohort was established prospectively and analyses were computed retrospectively.

Briefly, eligible patients were all adults with advanced NSCLCs after failure of at least one line of platinum‐based chemotherapy. Patients were included between September 1, 2015 and September 30, 2016, when nivolumab was started. Nivolumab (3 mg/kg of body weight) was infused every 15 days until DP (RECIST criteria v1.1^19^) or unacceptable toxicity, according to the National Cancer Institute Common Terminology Criteria for Adverse Events (CTCAE) v4.0.^20^ Determination of the PD‐L1–expression level was not required. Patients with epidermal growth factor receptor (*EGFR*) mutations or anaplastic lymphoma kinase (*ALK*) translocations were eligible if they had received prior targeted tyrosine kinase inhibitor therapy and at least one line of platinum‐based chemotherapy. They could have brain metastases, active or not and pretreated or not. Exclusion criteria were prior participation in an ICI trial or refusal to participate.

Database lock was on December 31, 2018. Patients alive ≥ 2 years after the first nivolumab infusion were defined as LTSs and their outcomes were analyzed. Three LTS groups were created according to the nivolumab‐administration duration: <2 years, with nivolumab discontinued for any cause; 2 years of therapy (clinical recommendations); and > 2 years, until DP or unacceptable toxicity. During the investigation period, patients meeting inclusion criteria were selected from each center's database. Database access was accorded to the first author in association with the referents of each participating center.

### 2.2 Study endpoints

Co‐primary endpoints were LTSs’ progression‐free survival (PFS) for 2‐ and > 2 years of nivolumab‐administration, defined as the time between starting nivolumab and tumor progression or death from any cause, with censoring of patients lost‐to‐follow‐up (ie, censored at last update), and OS for the three groups, defined as the time from nivolumab onset to death from any cause, censored at last update for survivors. The focus was to try to discern an optimal treatment duration. Secondary endpoints were; ORR, according to RECIST criteria v1.1, evaluated by the investigators at each center and analyzed as intention‐to‐treat; LTSs’ clinical characteristics, treatment safety and tolerability, according to CTCAE^20^; and LTSs’ survival.

### 2.3 Statistical analysis

Efficacy and safety were assessed for all included patients who received one or more nivolumab dose(s). This update represents a minimum of 24‐month follow‐up analysis. Continuous variables are expressed as the number of not missing data, median [range] maximum. Categorical variables are expressed as the total number (%) per category. For survival analysis, Kaplan‐Meier estimates were plotted and medians with their 95% confidence intervals (CIs). Survival data according to treatment duration were compared using a log‐rank test. Objective response rates was defined as the percentage of patients achieving partial (PR) or complete response (CR) to nivolumab. All statistical analyses were computed with SAS^®^ software (SAS Institute) v9.4 on Windows™. Statistical significance was set at *P* < .05.

## 3 RESULTS

### 3.1 Patient characteristics

Between September 1, 2015 and September 30, 2016, 259 all‐comer, consecutive patients were enrolled in the initial study.^13^ At database lock, 65 (25%) patients were defined as LTSs and included for analysis. The < 2‐, 2‐, and > 2‐year nivolumab‐administration groups comprised, respectively: 47, 7, and 11 LTSs. Their characteristics are given in Table 1. Baseline characteristics of the 65 patients who survived ≥ 2 years closely resembled those of the entire cohort. LTSs’ median age was 59 years, 73.8% were men, all NSCLCs were metastatic, 63.1% were adenocarcinomas and 33.8% had brain metastases at nivolumab onset. They had received a median of 1 [1‐6] previous systemic therapy lines. All 2‐ and > 2‐year LTSs were prior or active smokers. PD‐L1 expression was not reported.

**Table 1:** Baseline characteristics of the 259 patients (pts) and 65 long‐term survivors (LTSs), according to nivolumab treatment duration

Characteristic	All pts (N = 259)	LTSs (n = 65)	Nivolumab treatment duration		
			<2 y (n = 47)	2 y (n = 7)	>2 y (n = 11)
Median age, y [range]	62 [29‐85]	59 [39‐80]	59 [45‐80]	52 [39‐72]	58 [53‐66]
Sex, n (%)					
Female	72 (27.8)	17 (26.2)	12 (25.5)	2 (28.6)	3 (27.3)
Male	187 (72.2)	48 (73.8)	35 (74.5)	5 (71.4)	8 (72.7)
Smoking history, n (%)	222 (90.6)	57 (87.7)	39 (83)	7 (100)	11 (100)
Histology, n (%)					
Adenocarcinomas	165 (63.7)	41 (63.1)	31 (66.0)	4 (57.1)	6 (54.5)
Squamous cell	70 (27.0)	15 (23.1)	11 (23.4)	3 (42.9)	1 (9.1)
Undifferentiated carcinomas	18 (6.9)	7 (10.8)	4 (8.5)	0 (0)	3 (27.3)
Others	6 (2.4)	2 (3.0)	1 (2.1)	0 (0)	1 (9.1)
Molecular alterations, n (%)					
*KRAS* mutation	55 (22.0)	14 (21.5)	8 (17.0)	3 (42.9)	3 (27.3)
*EGFR* mutation	11 (4.4)	1 (1.5)	1 (2.1)	0 (0)	0 (0)
*BRAF* mutation	4 (1.6)	3 (4.7)	2 (4.3)	0 (0)	1 (9.1)
Other or not found	189 (72)	47 (72.3)	36 (76.6)	4 (57.1)	7 (63.6)
Number of prior lines, median [range]	1 [1‐6]	1 [1‐6]	1 [1‐6]	1 [1‐2]	1 [1‐4]
BMs before nivolumab onset, n (%)	77 (29.7)	22 (33.8)	14 (29.8)	3 (42.9)	5 (45.5)
Median nivolumab treatment duration (mo)	2.3	14.5	10.6	24.2	32.3
Median time from diagnosis to nivolumab onset (mo)	9.8	10.9	11.3	12.8	8.1

Abbreviations: BMs, brain metastases; *BRAF*, v‐*RAF* murine sarcoma viral oncogene homolog B; *EGFR*, epidermal growth factor receptor; *KRAS*, Kirsten rat‐sarcoma viral oncogene.

### 3.2 Survival

**Figure 1:**
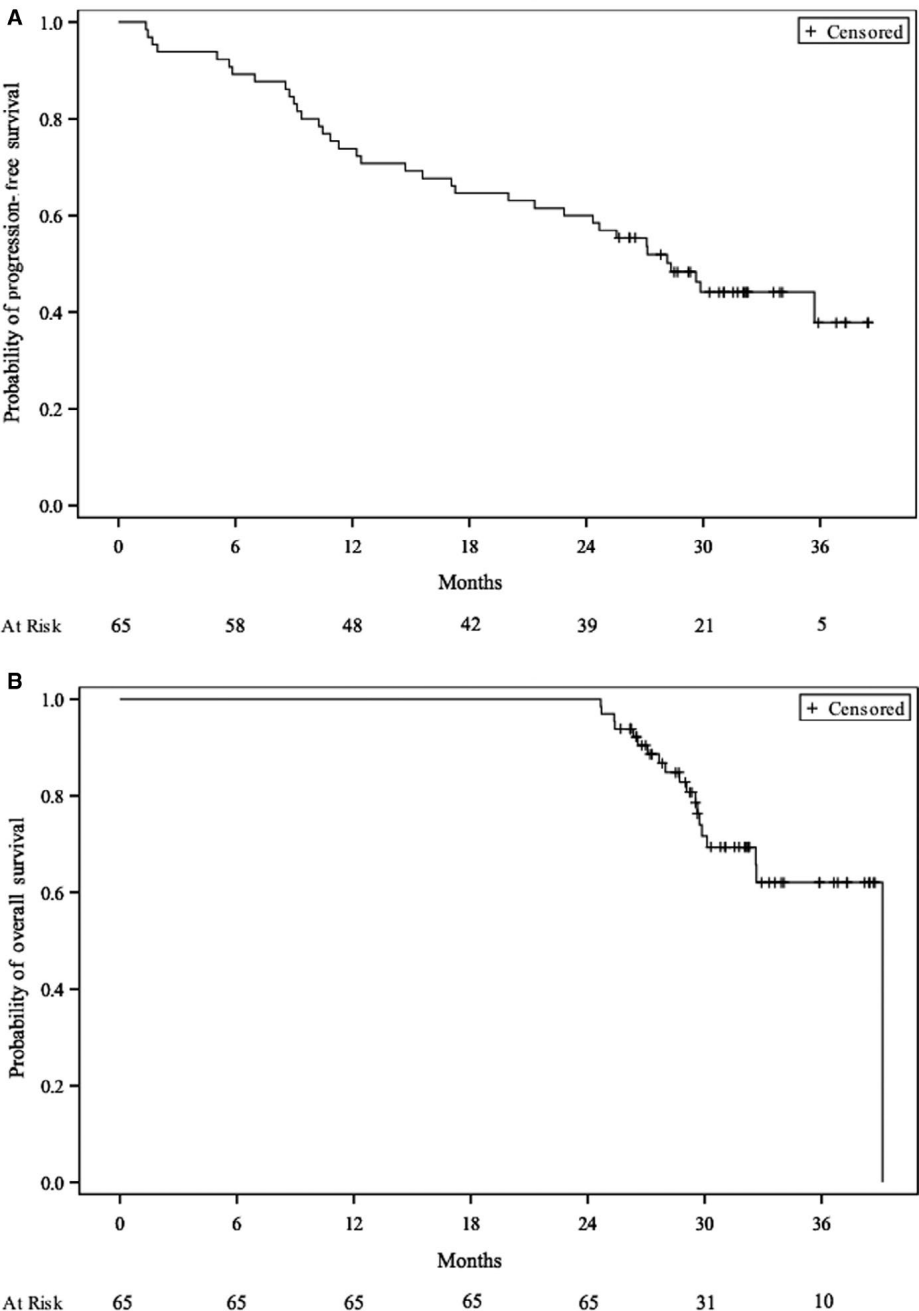
Kaplan‐Meier estimates of the probability of (A) progression‐free survival and (B) overall survival for the 65 long‐term survivors

The median follow‐up of the 259‐patient cohort was 32 (95% CI, 31.0‐34.0) months, median OS (mOS) was 9.7 (95% CI, 8.2‐11.0) months, and the Kaplan‐Meier estimated probability of 3‐year OS was 16.6%. LTSs’ median PFS (mPFS) was 28.4 months (95% CI, 21.4 to not reached [NR]) and mOS was not reached (95% CI, 32.6 to NR). At 30 and 36 months, respectively, PFS rates were 44.1% and 37.8%, with OS at 71.7% and 62.1% (Figure 1).

For the < 2‐year group, mPFS lasted 20.0 (95% CI, 11.3‐28.2) months and mOS 32.7 (95% CI, 29.7 to NR) months. Their 24‐month PFS was 44.7%; both PFS rates were 30.4% at 30‐ and 36‐months, with respective OS rates of 63% and 49%.

For the 2‐year group, mPFS and mOS were not reached. Their 24‐month PFS rate was 100%; with respective 30‐ and 36‐month PFS and OS rates of 71.4% for both, and 85.7% for both.

**Figure 2:**
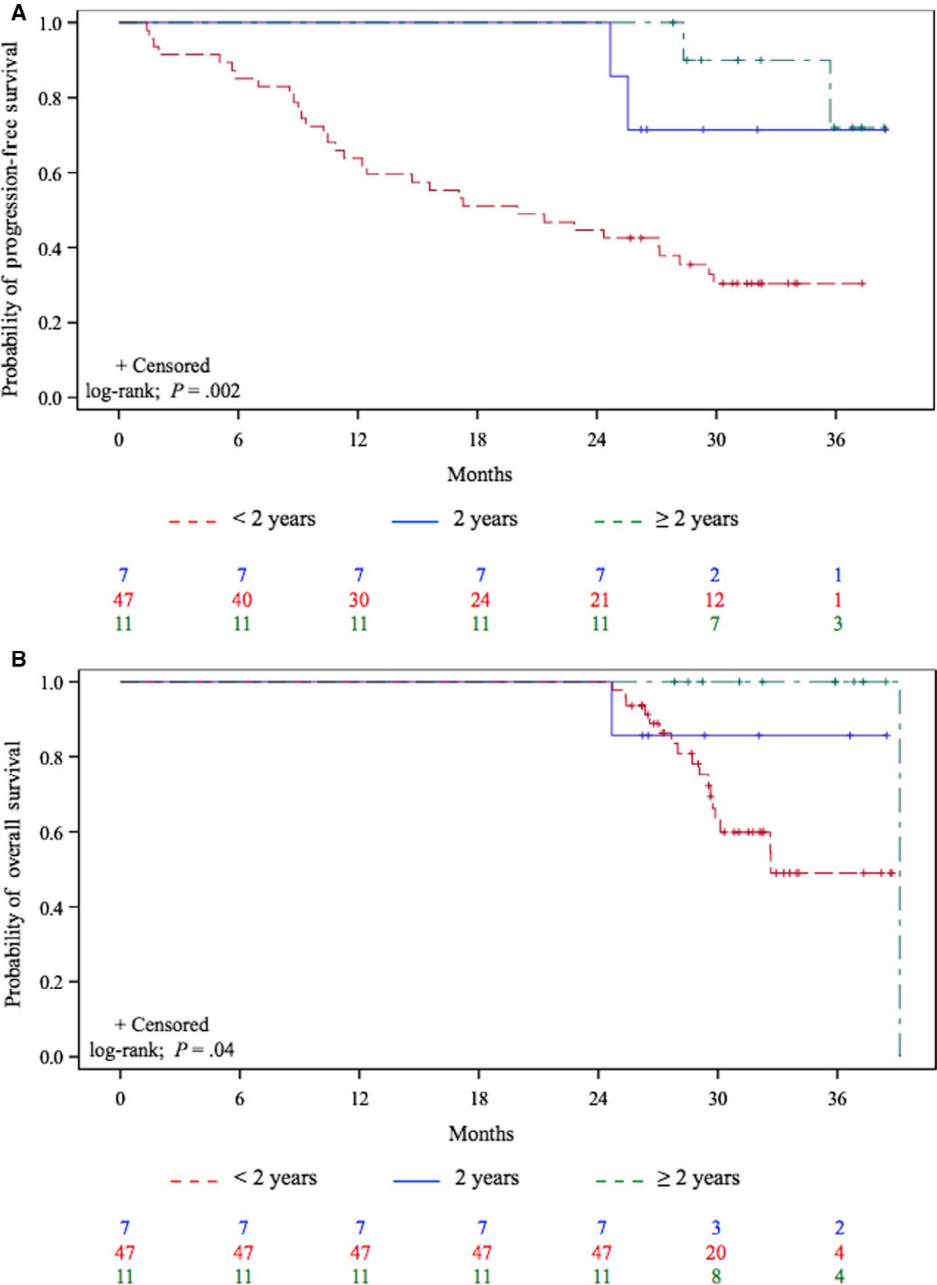
Kaplan‐Meier estimates of the probability of (A) progression‐free survival and (B) overall survival for the 65 long‐term survivors according to nivolumab treatment duration

For ≥ 2‐year group, mPFS and mOS were not reached. Their 24‐, 30‐ and 36‐month PFS rates were 100%, 90%, and 72%, respectively, with 30‐ and 36‐month Kaplan‐Meier estimated probability of OS rates both 100% (Figure 2).

**Figure 3:**
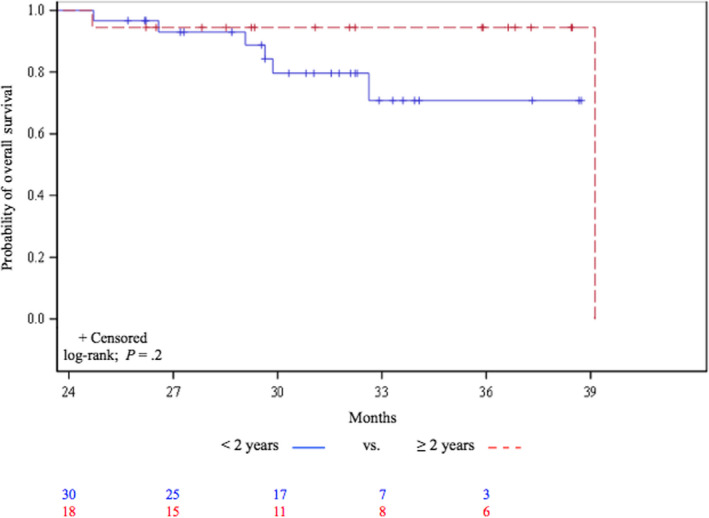
Kaplan‐Meier estimates of the probability of overall survival among long‐term survivors with progression‐free survival ≥ 12 mo, according to nivolumab administration duration

Using a log‐rank test, survival was significantly longer for the 2‐ and > 2‐year groups (*P* < .05). To further examine that finding, we compared group survival rates of patients with < 2 years vs those with 2 and > 2 years of nivolumab‐administration; only patients with PFS ≥ 1 year were included (*P* = .2) (Figure 3).

### 3.3 Response

LTSs’ ORR was 61.6% with 10.8% CRs and 50.8% of PRs. ORRs for the < 2‐, 2‐, and > 2‐year groups, respectively, were 51%, 85.7%, and 90.9%. Detailed nivolumab responses are reported in Table 2. Among the 259 cohort patients, 7 achieved CRs and all were LTSs,^13^ 5 from the < 2‐year group and 1 each from the 2‐ or > 2‐year group. Thirty‐three cohort patients had PRs.

**Table 2:** Detail of best nivolumab responses

Responses	LTSs	Nivolumab treatment duration		
	(N = 65)	<2 y (n = 47)	2 y (n = 7)	>2 y (n = 11)
Complete response, n (%)	7 (10.8)	5 (10.6)	1 (14.3)	1 (9.1)
Partial response, n (%)	33 (50.8)	19 (40.4)	5 (71.4)	9 (81.8)
Stable disease, n (%)	21 (32.3)	19 (40.4)	1 (14.3)	1 (9.1)
Progressive disease, n (%)	4 (6.2)	4 (8.5)	0 (0)	0 (0)
Not assessable, n (%)	0 (0)	0 (0)	0 (0)	0 (0)
Objective response rate (%)	61.6	51	85.7	90.9

Note

Results are expressed as n (%).

Abbreviation: LTSs, long‐term survivors.

### 3.4 Safety outcomes

**Figure 4:**
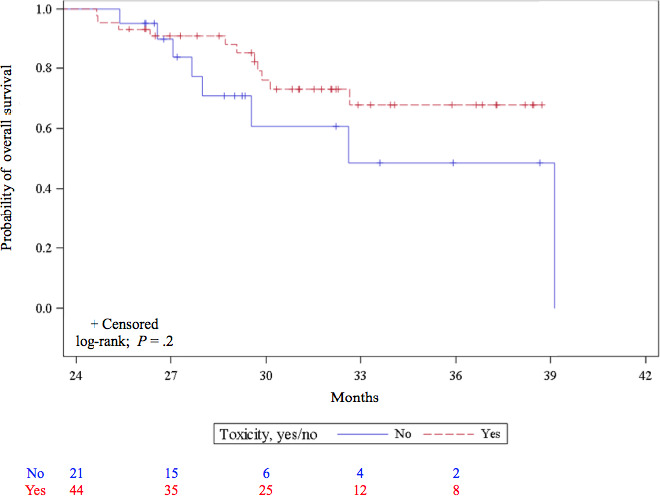
Kaplan‐Meier estimates of the probability of overall survival for the 65 long‐term survivors according to any‐grade adverse event occurrence

The rate of any‐grade treatment‐related immune adverse events (AEs) was 40.9% for the 259 treated cohort patients and it rose to 67.7% for LTSs, with 26.6% grade > 2 (Table 3). Occurrence of any‐grade AEs did not significantly impact OS of LTSs (*P* = .2) (Figure 4). Any‐grade toxicity rates for patients from the < 2‐, 2‐, and > 2‐year groups, respectively, were: 63.8%, 85.7%, and 72.7%, with 34.8%, 0%, and 9.1% grade > 2. Among the 5 patients with CRs who received nivolumab for < 2 years, 3 experienced grade‐3 AEs and 2 developed grade‐2 AEs. The other 2 CRs also had grade‐2 AEs.

**Table 3:** Safety Profile

Toxicity	LTSs	Nivolumab treatment duration		
	(N = 65)	<2 y (n = 47)	2 y (n = 7)	>2 y (n = 11)
Any grade	44 (67.7)	30 (63.8)	6 (85.7)	8 (72.7)
Grade > 2	17 (26.2)	16 (34.0)	0 (0)	1 (9.1)

Note

Results are expressed as n (%).

Abbreviations: LTSs, long‐term survivors.

### 3.5 Reasons for discontinuation and outcomes

Twenty‐three < 2‐year treated patients experienced DP and 14 unacceptable toxicity. For 10 of them, the treating physician decided to stop nivolumab. That decision (usually after 12 or 18 months) depended on local practices and/or patient's preferences and lifestyle. Physicians stopped nivolumab for 6 2‐year group patients and another died of an unknown cause. The ≥ 2‐year group experienced two DPs and one unacceptable toxicity, while physicians stopped treatment for 4 patients after 2 years; 4 patients were still receiving nivolumab. At the cutoff date, 28 (43.1%) patients had not experienced DP: 14, 5, and 9 patients from the < 2‐, 2‐, and > 2‐year groups, respectively. Subsequent therapies after nivolumab are summarized in Table 4.

**Table 4:** Subsequent therapies after nivolumab

Subsequent therapies after nivolumab	Nivolumab treatment duration		
	<2 y	2 y	>2 y
	n = 25	n = 1	n = 1
Carboplatin/ gemcitabine or paclitaxel/ ± bevacizumab	6 (24.0)	—	—
Paclitaxel/ bevacizumab	3 (12.0)	—	—
Pemetrexed/ bevacizumab	1 (4.0)	—	—
Paclitaxel or gemcitabine or pemetrexed	4 (16.0)	—	—
Docetaxel	3 (12.0)	—	—
Rechallenge nivolumab	2 (8.0)	1 (100)	—
Targeted therapy^a^	3 (12.0)	—	—
Other	1 (4.0)	—	—
Missing data	2 (8.0)	—	1 (100)

Note

Results are expressed as n (%).

^a^ erlotinib (n = 2), afatinib (n = 1).

## 4 DISCUSSION

Based on a real‐life, prospective cohort of 259 patients, the results of this study highlight the survival of advanced NSCLC LTSs at least 2 years after starting nivolumab. The 36‐month PFS and OS rates were 9.7% and 16.6%, respectively, consistent with the recently reported 3‐year OS rates.^18^, ^21^, ^22^ Published 5‐year OS rates are also in agreement with our findings.^16^, ^23^ Indeed, 25% of our entire cohort were LTSs and their baseline characteristics were mostly comparable to those of all treated patients. LTSs achieved 36‐month PFS and OS rates of 37.8% and 62.1%, respectively. Survival was prolonged for each of the three LTS subgroups. Our observations favor continuing immunotherapy beyond 2 years, with a trend toward better survival. Finally, all patients with CRs were LTSs; they had no signs of disease relapse at database lock.

Our main objective of study was to try to discern an optimized nivolumab‐administration duration for pretreated, advanced NSCLC patients. This aim is particularly relevant because optimal ICI treatment duration remains unknown and no strong data have been reported. Some clinical trial protocols planned a specified treatment duration and health authorities also recommended pursuing immunotherapy until DP or up to 2 years for patients without DP.^5^, ^24^ However, the authors of published studies did not precisely address optimizing ICI treatment duration for NSCLCs or in other tumor types.

Herein, we noted a trend favoring ICI continuation beyond 2 years with higher 3‐year OS rates (85.7% and 100% for the 2‐ and > 2‐year groups, respectively), and neither group reached mOS. Focusing on the < 2‐year group, survival rates did not seem to favor nivolumab discontinuation before 2 years, as they had a lower 3‐year OS rate of 49% and mOS at 32.7 months. However, these results must be interpreted with caution, given the very small numbers of patients, some of whom had stopped nivolumab before 2 years because of DP, making it impossible to analyze potential confounding factors (eg, performance status, tumor stage, and prior radiotherapy) and comparisons were not adjusted to them.

In CheckMate‐153, Spigel et al compared continuous nivolumab vs a fixed 1‐year treatment period.^25^ Among the patients still on nivolumab at 1 year, those treated continuously had significantly prolonged PFS, also with a trend toward longer OS. The authors suggested pursuing nivolumab beyond 1 year, but their study was not designed to answer this specific question. The 5‐year follow‐up data from CA209‐003 suggested patients may achieve long‐term survival with ≤ 2 years of nivolumab.^23^, ^26^ However, other approved ICIs require continuous administration until unacceptable toxicity or DP. Recent results of the phase Ib, Keynote‐001 trial demonstrated the 5‐year long‐term benefit of pembrolizumab.^22^ Patients who achieved PRs or SD after 2 years of pembrolizumab could stop treatment and then resume it if DP occurred. However, no subgroup details or characteristics encouraging continuation beyond 2 years were given. Finally, long‐term results of pivotal, phase III trials on ICIs for NSCLCs failed to provide clear answers.^14^, ^15^, ^17^, ^18^

The gaps in data are partially filled by results from studies on other cancers. For metastatic melanoma, the concept of treating until DP does not always apply. Indeed, many patients’ tumors responded to ICIs for years. Clinical trial results provided no evidence that > 2 years of checkpoint blockade was needed, especially for patients achieving CR or PR. Moreover, for patients obtaining CRs who had been treated for > 6 months, the risk of relapse after stopping treatment was low.^27^

We also wondered whether the best tumor response could predict survival, as previously described.^23^ The durability of responses achieved on nivolumab appeared to be an important contributor to OS. In our study, only seven patients achieved CRs with long‐term survival and no sign of disease relapse at database lock. Among patients who received ≥ 2 years of nivolumab, >85% had objective responses, and their 3‐year OS rate exceeded 85%. These findings are consistent with results of the Keynote‐001 and −010 trials, in which the ORR and 3‐year OS rates were high among patients who completed 2 years of treatment.^16^, ^17^ Patients whose tumors achieved PRs or SD as best response were apparently at higher risk for DP after stopping therapy, and defining optimal treatment duration for such patients deserves further study. Pertinently, however, baseline characteristics did not differ between surviving responders at database lock vs nonresponders (in an exploratory analysis).

It was previously described that ICI‐induced AE occurrence could influence response and survival.^23^, ^28^, ^29^, ^30^ The 67.7% AE rate for our LTSs was higher than for the entire cohort.^13^ AEs were less frequent but more severe for the < 2‐year group. In fact, patients treated < 2 years had 34.8% severe AEs, which might have affected their outcomes. For the 2‐ and ≥ 2‐year groups, we noted that nivolumab‐related AEs were more frequent among patients with therapeutic responses than those without. Nevertheless, any‐grade AE occurrence did not affect survival (*P* = .2).

The main limitation of our study was its design with relatively short follow‐up. Because PD‐L1 expression was unknown (not required in current practice), its predictive value could not be evaluated and, therefore, identifying any potential association between PD‐L1 tumor‐proportion score and survival is beyond the scope of this analysis. Furthermore, it seems highly probable that patients who continued the treatment beyond 2 years were those with the best clinical benefit.

## 5 CONCLUSION

Nivolumab provided long‐term benefit in real‐life practice with estimated 3‐year OS at 16.6% for pretreated, advanced NSCLC patients, with 25% LTSs. Nevertheless, optimal ICI‐administration duration for NSCLC has still not been established and prospective, randomized trials with adequate statistical design are needed.

## DISCLOSURE

The authors have stated that they have no conflicts of interest.

## CONFLICT OF INTEREST

The authors confirm that there are no known conflicts of interest associated with this publication and there has been no significant financial support for this work that could have influenced its outcome.

## AUTHORS CONTRIBUTIONS

All persons who meet the authorship criteria are listed as authors, and all authors certify that they have participated sufficiently in the work to take public responsibility for the content, including participation in the concept, design, analysis, writing, or revision of the manuscript. Furthermore, each author certifies that this material or similar material has not been and will not be submitted to or published in any other publication before its appearance in the *Cancer Medicine*.

***Category 1***

‐ Conception and design of study: M. Geier, G. Robinet.

‐ Acquisition of data: M. Geier, R. Descourt, R. Corre, G. Léveiller, R. Lamy, É. Goarant, JL Bizec, C. Bernier, G. Quéré.

‐ Analysis and/or interpretation of data: M. Geier, G. Robinet, E. Burte, C. Chouaid.

***Category 2***

‐ Drafting the manuscript: M. Geier, G. Robinet, C Chouaid, K. Amrane, E. Gaye, F. Lucia.

‐ Revising the manuscript critically for important intellectual content: All authors.

***Category 3***

‐ Approval of the version of the manuscript to be published (the names of all authors must be listed): M. Geier, R. Descourt, R. Corre, G. Léveiller, R. Lamy, É. Goarant, JL Bizec, C. Bernier, G. Quéré, K. Amrane, E. Gaye, F. Lucia, E. Burte, C. Chouaid, G. Robinet.

## STUDY OVERSIGHT

This noninterventional study was approved by a regional Ethics Committee and France's National Data Protection Authority (CNIL), in accordance with French law. All patients or their legal representatives provided written informed consent before enrollment.

## Data Availability

The datasets generated and/or analyzed during the current study are not publicly available but are available from the corresponding author on reasonable request.
